# 4-Phenyl Butyric Acid (4-PBA) Suppresses Neutrophil Recruitment in a Murine Model of Acute Perinatal Inflammation

**DOI:** 10.1155/jimr/2438058

**Published:** 2025-07-07

**Authors:** Christian Gille, Maylis Jungwirth, Silvia Pezer, Stefanie Dietz-Ziegler, Natascha Köstlin-Gille, Trim Lajqi

**Affiliations:** ^1^Department of Neonatology, Medical Faculty Heidelberg, University of Heidelberg, Heidelberg, Germany; ^2^Department of Neonatology, University of Tübingen, Tübingen, Germany

**Keywords:** 4-PBA, cytokines, inflammation, migration, neutrophil recruitment, neutrophils

## Abstract

Neutrophils are the first immune cells to be recruited to the site of infection and deregulated activation is linked to adverse outcome, especially in premature neonates. Dampening neutrophil activity may therefore be a means of preventing acute and chronic inflammatory diseases; however, little is known about potential drugs to modulate neutrophil activity. 4-Phenyl butyric acid (4-PBA) is a clinically used drug, which acts as a chemical chaperone to inhibit endoplasmic reticulum (ER) stress and to suppress immune activation. Here, we investigated the potential of 4-PBA to regulate neutrophil-mediated inflammation and specifically the recruitment cascade of neutrophils. We found that 4-PBA suppressed perinatal neutrophil recruitment cascade as assessed by fetal intravital microscopy (IVM), as well as transmigration of neutrophils through the endothelial compartment via the inositol-requiring enzyme (IRE)-1α/extracellular signal-regulated kinase (ERK) 1/2 signaling pathway. Likewise, 4-PBA promoted an anti-inflammatory phenotype by suppressing the release of pro-inflammatory mediators in bone marrow neutrophils and endothelial cells in vitro. Taken together, our data indicate that 4-PBA can exert anti-inflammatory effects by limiting excessive neutrophil infiltration into inflamed tissues, thereby holding significant therapeutic potential in managing various inflammatory diseases.

## 1. Introduction

Acute inflammation is a critical immune reaction that supports defense mechanisms against infections and injury by facilitating tissue repair and eliminating the invading intruders. However, sustained excessive or inappropriate inflammation driven by immune cells and inflammatory mediators is the cause of various chronic inflammatory disorders.

Neutrophils (also known as polymorphonuclear neutrophils, PMNs), as a part of the innate immune system, represent one of the most crucial cells that start to appear during gestational week (GW) 8 in humans and reaching the peak of expansion during birth [1]. PMNs, as fully differentiated short-lived effector cells, act as the initial defense against pathogens, utilizing a range of antimicrobial agents, such as cytokines, chemokines, enzymes, and reactive oxygen species (ROS) [2, 3]. They have been considered as obsolete effector cells with limited functions during acute inflammation [4, 5]. However, a rising number of studies reveal that neutrophils are involved in various inflammatory reactions [2, 6]. Neutrophils are the primary cells to be recruited to the site of infection [7, 8]. Extravasation of neutrophils is initiated by inflammatory mediators released from tissue-resident cells encountering conserved pathogen-associated molecular patterns (PAMPs) from various pathogens [4, 5, 7]. The recruitment cascade of neutrophils includes several steps like tethering, rolling, adhesion, crawling, and transmigration [2, 7, 9], while the endothelial compartment can be conjointly activated to support neutrophil extravasation through the release of chemoattractants (i.e., monocyte chemoattractant protein-1 [MCP-1]) [10, 11]. The fine-tuning of the multistep neutrophil recruitment cascade is crucial for the inflammatory process, ensuing an appropriate response against invading intruders (i.e., microbes) [8, 12]. In the case of dysregulated activation, neutrophils greatly contribute to adverse outcome of systemic inflammatory response syndrome (SIRS) [13].

The endoplasmic reticulum (ER) serves many pivotal roles in maintaining cellular homeostasis by facilitating protein synthesis and folding, calcium storage as well as lipid synthesis [14, 15]. Persistent disruption of normal ER functions or extensive accumulation of misfolded proteins results in a cellular reaction known as ER stress, characterized by increased activation of the unfolded protein response (UPR), which promotes inflammatory signaling pathways, thus contributing to the development of numerous chronic inflammatory conditions [16, 17]. 4-Phenyl butyric acid (4-PBA) is a small molecule known for its immune-suppressive anti-inflammatory properties [18, 19]. Several studies have shown that 4-PBA serves as a potent inhibitor of histone deacetylase activity (HDAC) and ER stress [20–22]. Furthermore, it shows anticancer activities against solid tumors and is used as an approved ammonia scavenger to treat urea cycle disorders [23, 24]. The role of 4-PBA suppressing LPS-induced inflammation through the regulation of ER stress characterized by declined levels of pro-inflammatory cytokines has been reported [19, 25]. Moreover, numerous studies have demonstrated that 4-PBA can limit the recruitment of mononuclear cells, especially monocytes resulting in reduced inflammation [25–27]. However, the role of 4-PBA in altering neutrophil recruitment and its impact on the inflammatory activation has not yet been fully resolved.

In the current study, we investigated the potential of 4-PBA to modulate neutrophil recruitment and exert anti-inflammatory effects. Our findings show that 4-PBA inhibits the perinatal neutrophil recruitment cascade, as assessed by intravital microscopy (IVM), and impairs neutrophil migration abilities in vitro. Furthermore, 4-PBA reduces pro-inflammatory mediators in both neutrophils and endothelial cells, with its anti-inflammatory effects on neutrophils mediated through alterations in the inositol-requiring enzyme (IRE)-1α/extracellular signal-regulated kinase (ERK) 1/2 signaling pathway.

## 2. Materials and Methods

### 2.1. Animals and IVM

#### 2.1.1. Animals

For IVM, LysEGFP reporter mice (C57BL/6N background, EGFP knocked into the neutrophil and monocyte-specific lysozyme M [lys] locus) were utilized [28]. C57BL/6J adult mice (3–6 months old) were used for the isolation of PMNs. Vaginal plug positive mice were classified E0.5 at noon for timed pregnancy experiments involving IVM. The mice were maintained on a 12-h dark/light cycle with access to food and water and experiments were conducted in accordance with the EU Guidelines (Directive: 2010/63/EU), and were approved by the regional authorities of the Karlsruhe Government Office, Germany (Ref. No. Az T-02/20, Az T-18/21 and AZ 35-9185.81/G-111/12).

#### 2.1.2. IVM

**Figure 1:**
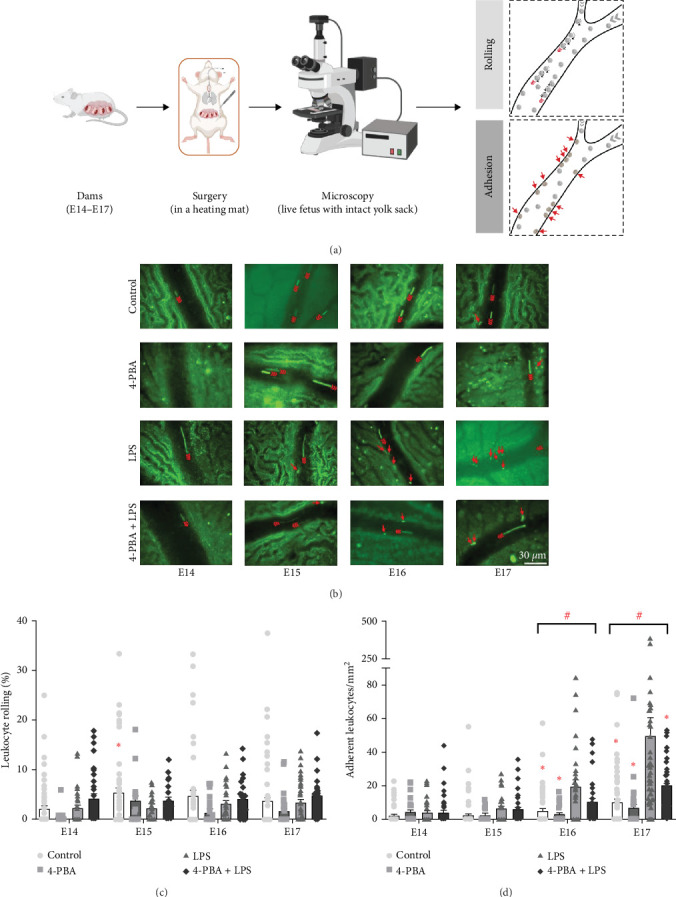
Impact of 4-PBA on leukocyte rolling and adhesion in vivo. (A) Schematic representation of the surgical preparation of mouse embryos for intravital microscopy (IVM) using an upright fluorescence microscope. Representative images of leukocyte recruitment (B), and their quantitative analyses of leukocyte rolling (C) and adhesion (D) were assessed in fetal mice across different gestational age groups. Mice were treated with either saline (0.9% NaCl; control, •), 4-PBA (■), LPS (▲), or a combination of 4-PBA and LPS (♦), as described. Data are given as mean + SEM, two-way ANOVA (Holm–Sidak post hoc), *n* = 3–17 experiments with 1–5 newborn mice per condition, where *⁣*^*∗*^*p* < 0.05 denotes a significant change versus LPS treatment, and #*p* < 0.05 represents a significant change versus E14 gestational age group.

LPS from *Escherichia coli* (055:B5, #tlrl-pb5lps, InvivoGen, Toulouse, France) was dissolved in saline (10 µg LPS/50 µL). 0.25 mg/kg LPS were injected twice intraperitoneally (i.p.) 20 h and 4 h prior to IVM. In the intervention group, 25 mg/kg 4-PBA (#P21005, Sigma–Aldrich, St. Louis, USA) was injected i.p. at the same time points prior to IVM. Control animals were administered with proportionate volumes of saline or 4-PBA alone. Mice showed no signs of physical distress upon LPS injection at either dose. Surgical preparation and IVM procedures were performed as described previously [29–31]. For anesthesia, dams were administered i.p. with 125 mg/kg ketamine (KetanestS, Pfizer Pharma GmbH, Berlin, Germany) and 12.5 mg/kg xylazine (Xylavet, CP-Pharma GmbH, Burgdorf, Germany) and placed on a heating pad. Upon achieving optimal anesthesia, a sub-laryngeal tracheal cannula was carefully inserted and secured with sutures. Anesthesia was maintained through a carotid artery catheter. A small abdominal incision was performed and one side of the uterus was exposed. After careful removal of the uterine wall opposite the placenta, a single viable fetus, still enclosed within an intact yolk sac (YS), was gently positioned on viscose gel atop a microscope stage. The preparation was continuously superfused with a 37°C bicarbonate-buffered saline solution and covered with a glass coverslip (Figure 1A).

IVM was performed with an upright microscope (LaVision BioTec GmbH, Bielefeld, Germany; Olympus BX51W1, Hamburg, Germany), featuring a 20x objective (UMPLFL20XW/0.50 U M Plan Fluorit, Zeiss, Germany). The imaging session was performed for up to 2 h per mouse using a CCD camera (LaVision Imager Compact QE). Rolling of EGFP-labeled cells was quantified as the rolling flux fraction, defined as the percentage of rolling cells relative to the total number of labeled cells passing through the vessel within 1 min. Adhesion was measured by counting the number of cells that adhered for more than 30 s per square millimeter of vascular surface area.

### 2.2. Isolation of Bone Marrow PMNs

Isolation of bone marrow PMNs was performed by density gradient centrifugation using LSM 1077 (#C-44010, PromoCell, Heidelberg, Germany) as described previously [29, 32, 33]. Briefly, femur and tibia from C57BL/6J wild type mice were released from connective tissue and processed further under sterile conditions. Following the removal of the bone epiphyses, the marrow was extracted by flushing with RPMI-1640 medium (#R8758, Sigma–Aldrich) containing 2 mM ethylenediaminetetraacetic acid (EDTA). The medium was enriched with 10% heat-inactivated, sterile-filtered FCS (#PB-FCS-EU-0500, PeloBiotech, Planegg, Germany), 100 IU/mL penicillin/100 µg/mL streptomycin (#P4333) and 2.5 μg/mL amphotericin B (#A2942) from Sigma–Aldrich. The resulting pellet was filtered through a 100 µm cell strainer, followed by a hypotonic erythrocyte lysis buffer (0.15 M NH_4_Cl, 0.01 M NaHCO_3_, and 0.001 M EDTA) for 3–5 min. Then, PMNs were isolated by density gradient centrifugation using Percoll (#17-0891-02, GE Healthcare Bio-Sciences, Uppsala, Sweden) from the 64%/81% interphase, subsequently washed twice by sterile buffered saline (DPBS; #D8537, Sigma–Aldrich), counted and processed for further experimental settings (stimulation, transmigration). The purity of neutrophil cell suspension was confirmed through flow cytometry, employing standard neutrophil markers as demonstrated previously (Figure S1A) [29].

### 2.3. Culture of Mouse Primary Lung Microvascular Endothelial Cells (mPLMECs)

C57BL/6 mPLMECs were obtained from Cell Biologics (#C57-6011, Chicago, IL, USA) and cultured at 37°C/5% CO_2_ in T75 culture flasks using EASY endothelial cell growth medium (#PB-MH-100-2100) supplemented with EASY growth supplement (#PB-SH-100-2100) and gentamycin-amphotericin (#PB-SX-000-0099) obtained from PeloBiotech. At 70%–90% confluence mPLMECs were washed carefully with sterile DPBS, and detached from the flasks using trypsin-EDTA (0.25% trypsin with 1 mM EDTA). A density of 100.000 mPLMECs per well was seeded in 12-well plates and stimulated according to the protocol detailed below.

## 3. In Vitro Cell Stimulation

Bone marrow PMNs (1 × 10^6^ cells/well) were isolated and immediately stimulated, along with mPLMECs, with 4-PBA (15 mM; #P21005, Sigma–Aldrich), LPS (100 ng/mL; *E. coli* serotype 055:B5, #tlrl-pb5lps, InvivoGen) or a combination of 4-PBA and LPS (pretreated for 1 h with 15 mM 4-PBA followed by treatment with 100 ng/mL LPS) for 12 h at 37°C with 5% CO_2_. Untreated control groups were maintained in complete media under the same conditions.

### 3.1. Antibodies

Primary antibodies were obtained from Cell Signaling (Danvers, MA, USA): phospho-p44/42 MAPK (ERK1/2) (Thr202/Tyr204) (#9106) and p44/42 MAPK (ERK1/2) (#9107). IRE-1α antibody (B-12; #sc-390960) was obtained from Santa Cruz Biotechnology (Heidelberg, Germany). β-actin antibody (#A5441, Sigma–Aldrich) was used as a loading control for western blotting. Secondary horseradish peroxidase (HRP)-conjugated antibodies, anti-rabbit (#111-035-144) and anti-mouse (#115-035-166), were purchased from Dianova (Hamburg, Germany).

### 3.2. Enzyme-Linked Immunosorbent Assay (ELISA)

Cell culture supernatants have been collected 12 h after the treatment and centrifuged at 10,000 x G for 5 min at 4°C. Samples were stored at −80°C until the measurement. The concentration of tumor necrosis factor (TNF)-α (#430901), interleukin (IL)-6 (#431301), IL-10 (#431411) and MCP-1 (#432701) were assayed using commercial kits acquired from BioLegend (San Diego, USA) as previously described [33–35]. Absorbance was recorded accordingly at 450/570 nm using an iMark microplate reader (Bio-Rad Laboratories, CA, USA).

### 3.3. Measurement of ROS

PMNs at a density 1 × 10^5^ cells per well were seeded in a clear bottom 96-well plate black plate and stimulated following a previously established protocol. The production of ROS was assessed using the cellular ROS s (DCFDA/H2DCFDA kit, #ab113851, Abcam, Cambridge, UK). After 12 h of stimulation, neutrophils were resuspended in a 20 µM solution of 2′, 7′-dichlorofluorescin diacetate (DCF-DA) and incubated for 30 min at 37°C in the dark. Postincubation, cells were washed (1x buffer) and subsequently resuspended in freshly prepared 1× supplemented buffer containing 0.1% FCS. The fluorogenic DCF-DA is internalized by viable cells, where intracellular esterases convert it into a nonfluorescent derivative. ROS-mediated oxidation then generates the highly fluorescent 2′, 7′-dichlorofluorescein (DCF). Fluorescence intensity was recorded using a PerkinElmer Wallac Victor3 microplate reader (PerkinElmer Life and Analytical Sciences, Turku, Finland) with an excitation wavelength of 485 nm and an emission wavelength of 535 nm.

### 3.4. Pierce Protein Assay for Protein Concentration

The concentration of protein lysates was measured using commercially available kits (Pierce 660 nm, #22662, Thermo Fisher Scientific, Waltham, USA) following manufacturer's instructions. Standards, samples and blanks were plated in culture 96-well plates and mixed with the protein reagent supplemented by IDCR (#22663, Thermo Fischer Scientific). After 5 min, absorbance was assessed at 660 nm using the iMark microplate reader.

### 3.5. Sodium Dodecyl Sulfate-Polyacrylamide Gel Electrophoresis (SDS-PAGE) Western Blotting

Bone marrow PMNs treated for 12 h were lysed in ice-cold radioimmunoprecipitation assay (RIPA) buffer (50 mM Tris–HCl (pH 8), 150 mM NaCl, 1% NP-40, 0.5% Na-deoxycholate, 0.1% SDS), supplemented with freshly prepared protease (100 mg/mL Pefabloc; 1 mg/mL Pepstatin A; 1 mg/mL Leupeptin) and phosphatase (10 mM sodium orthovanadate) inhibitors [36–38]. The lysates were vortexed briefly, incubated on ice for 1 min to ensure complete cell disruption, and then centrifuged at 12,000 × *g* for 30 min at 4°C to remove cellular debris. The supernatants were collected and stored at −80°C until further use. Protein samples were prepared by mixing supernatants with 5× Laemmli buffer and heated at 95°C for 5 min to denature the proteins and reduce disulfide bonds. Proteins were resolved via 10% SDS-PAGE and transferred onto a 0.45-μm polyvinylidene fluoride (PVDF) membrane. To prevent nonspecific binding, the membrane was blocked with 1% BSA in TBS-T (Tris-buffered saline with 0.1% Tween-20) for 30–45 min at room temperature, and incubated with primary antibodies overnight at 4°C. After primary antibody incubation, the membrane was washed with TBST to remove excess antibody, and incubated with HRP-conjugated secondary antibodies for 1 h at room temperature. Afterwards the membrane was washed again with TBST, and the protein bands were detected using enhanced chemiluminescence (ECL) reagents. Protein bands were visualized using a Chemi-Doc XRS + imaging system (Bio-Rad Laboratories). The captured images were analyzed and quantified using Image Lab Ver. 6.0.1 software (Bio-Rad Laboratories, Hercules, CA, USA).

### 3.6. Complementary DNA Isolation and RT-PCR

Six hours after the treatment, total RNA from PMNs and mPLMEC samples was extracted using TRIsure Lysis Reagent as previously demonstrated [39]. The concentration and quality of total RNA were evaluated with a Nanodrop DS11 FX+ (DeNovix, Wilmington, USA). cDNA synthesis was performed using the high capacity cDNA reverse transcription kit (#4368814, Applied Biosystems). mRNA expression of target genes was analyzed by real-time quantitative PCR (qPCR) using a StepOnePlus system (Applied Biosystems, Waltham, USA). The following primer pairs and their sequences were used: TNF-α (forward: CTGTAGCCCACGTCGTAGC; reverse: TTGAGATCCATGCCGTTG), IL-6 (forward: CCTCTCTGCAAGAGACTTCCATCCA; reverse: GGCCGTGGTTGTCACCAGCA), MCP-1 (forward: CATCCACGTGTTGGCTCA; reverse: GATCATCTTGCTGGTGAATGAGT), P-selectin glycoprotein ligand (PSGL)-1 (forward: CAGAGACCTCAAAACCAGCACC; reverse: GGTAGGTTCTGTGGAAGGGACT), CD11a (forward: AGATCGAGTCCGGACCCACAG; reverse: GGCAGTGATAGAGGCCTCCCG), E-Selectin (forward: GGACACCACAAATCCCAGTCTG; reverse: TCGCAGGAGAACTCACAACTGG), P-Selectin (forward: AAGATGCCTGGCTACTGGACAC; reverse: CAAGAGGCTGAACGCAGGTCAT), ICAM-1 (forward: AAACCAGACCCTGGAACTGCAC; reverse: GCCTGGCATTTCAGAGTCTGCT), and GAPDH (forward: CATCACTGCCACCCAGAAGACTG; reverse: ATGCCAGTGAGCTTCCCGTTCAG). GAPDH served as the housekeeping gene and the relative gene expression was determined using the comparative *C*_T_ (2^−ΔΔCT^) method [40].

### 3.7. Transmigration

In vitro transmigration of PMNs was performed using Corning HTS Transwell 96-well permeable transmigration chambers (#CLS3388, Sigma–Aldrich), as described [32]. Leukocytes were pretreated with or without 4-PBA (15 mM) for 1 h. A negative control (1x HBSS buffer, #14025050, Life Technologies, Paisley, UK) and a positive control (cells subjected to an apical gradient of CXCL1/KC [1 µg/mL, #250-11, Peprotech, Hamburg, Germany]) were used for 2 h at 37°C/5% CO_2_. Fluorescent calcein (#C1430, Invitrogen, Waltham, USA) was used to stain the transmigrated cells, followed by incubation for 45 min at 37°C in a 5% CO_2_ environment. After staining, cells were washed by HBSS supplemented with 0.5% bovine serum albumin (BSA), and lysed using cetyltrimethylammonium bromide (CTAB) buffer (#9161.1, Carl Roth, Karlsruhe, Germany). Fluorescence was detected using a PerkinElmer Wallac Victor3 microplate reader at 485 nm excitation and 535 nm emission.

### 3.8. Blood Plasma Isolation and Cytokine Analysis

C57BL/6 mice were administered 0.9% NaCl (control), 4-PBA (25 mg/kg), LPS (0.25 mg/kg), or a combination of 4-PBA (25 mg/kg) pretreatment followed by LPS (0.25 mg/kg) for 24 h. Blood samples were drawn using heparinized needles into 1.5 mL heparin-coated tubes, followed by centrifugation at 2000 × *g* for 10 min at room temperature. Then, the plasma samples were collected immediately and stored at −80°C until further analysis. Cytokine production has been determined using ELISA kits as described above, and the results were normalized to the protein content of each plasma sample.

### 3.9. Analysis of Cell Cytotoxicity and Viability

To assess the cytotoxicity of the stressors 4-PBA and LPS, we used the colorimetric Cell Cytotoxicity Assay Kit (#ab112118, Abcam, Cambridge, UK). 10.000 cells per well were plated in a 96-well plate and treated as described previously. After 12 h of treatment, 20 µL of assay solution was added to each well, and the plate was incubated at 37°C with 5% CO_2_ for 4 h, protected from light. Absorbance was measured at 570 nm using an iMark microplate reader, with data presented as relative cytotoxicity (control set to 100%) (Figure S1B).

Viability of PMNs following single or combined stimulation with 4-PBA and LPS was assessed using the MTT assay. PMNs (2 × 10^5^ cells/well) were plated in a 96-well plate, treated for 12 h, and then incubated with 10 µL/well of 0.5 mg/mL MTT solution for 4 h at 37°C and 5% CO_2_. After incubation, 100 µL of solubilization solution was added, and the plate was incubated overnight. Absorbance was measured at 570 nm using an iMark microplate reader, with data presented as relative viability (control assigned as 100%) (Figure S1C).

### 3.10. Statistics

GraphPad Prism 8.0.2 (GraphPad Software, San Diego, USA) and SigmaPlot Software Ver. 12.0 (Systat Software GmbH, Erkrath, Germany) were used for statistical analysis and graph creation. The normality of the data was assessed using the Shapiro–Wilk test. If not normal distributed, a nonparametric Kruskal–Wallis test was used, with the Benjamini–Hochberg FDR correction to control false positives. For parametric data, statistical comparisons were made using either one-way or two-way analysis of variance (ANOVA), followed by Holm–Sidak post hoc testing. Data are given as mean + SEM, with a *p*-Value of <0.05 considered statistically significant.

## 4. Results

### 4.1. 4-PBA Inhibits the Recruitment Cascade of Neutrophils In Vivo

Previous studies have demonstrated that 4-PBA serves as an important ER stress inhibitor with notable anti-inflammatory properties. These effects contribute to an immune-resolving reprograming of monocytes, characterized by diminished recruitment capabilities. To investigate whether 4-PBA similarly modulates neutrophil recruitment, we performed IVM experiments on fetal YS vessels (Figure 1).

Our results suggest that 4-PBA treatment tends to inversely affect the fraction of rolling leukocytes, although the change is not statistically significant (Figure 1B, C). Concomitantly, 4-PBA significantly reduces LPS-induced neutrophil adherence in E16 and E17 gestational stages in vivo (Figure 1B, D). Moreover, we observed a gestational age (GA)-dependent modulation of adhesive properties in fetal leukocytes, with E16 (*p*=0.041) and E17 (*p*  < 0.001) showing marked differences compared to the E14 group, emphasizing the crucial role of developmental stage in regulating recruitment mechanisms in vivo (Figure 1B, D).

To further elucidate the immunomodulatory role of 4-PBA, we assessed the systemic cytokine pattern profile in LPS-challenged mice. ELISA measurements of blood plasma of adult mice revealed that simultaneous administration of LPS and 4-PBA led to a distinct cytokine response. Notably, 4-PBA suppressed TNF-α production, consistent with its anti-inflammatory effects (Figure S2A). However, IL-6 and MCP-1 levels were markedly increased compared to LPS alone, indicating a selective pro-inflammatory response at the systemic level (Figure S2B,C). Interestingly, IL-10 levels remained unchanged compared to LPS treatment (Figure S2D).

### 4.2. 4-PBA Triggers Altered Inflammatory Responses in Murine Neutrophils in Vitro

As blood plasma serves as a dynamic “reservoir” enriched with inflammatory mediators secreted by various immune cells, we elaborated whether 4-PBA treatment would exert a distinct modulatory effect on the cytokine profile of PMNs in vitro.

**Figure 2:**
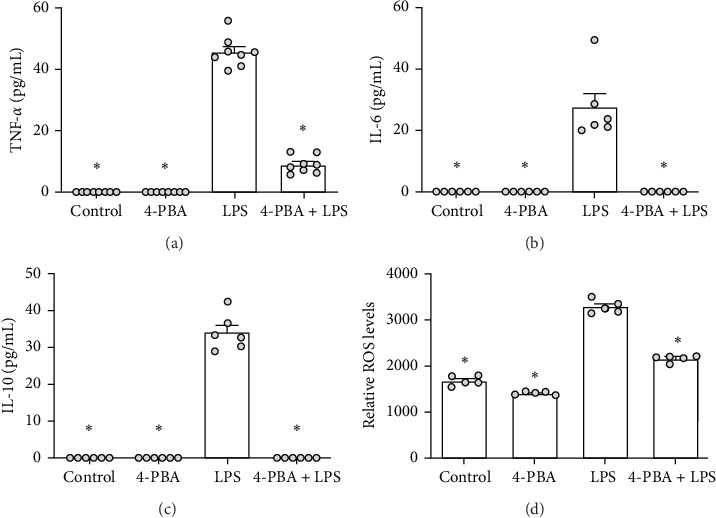
Effects of 4-PBA on the cytokine profile of bone marrow-derived PMNs in vitro. Bone marrow-derived PMNs were stimulated with 4-PBA, LPS, or a combination of 4-PBA and LPS for 12 h, as described. Supernatants were collected, and the cytokine production of TNF-α (A, *n* = 8), IL-6 (B, *n* = 6), and IL-10 (C, *n* = 6) was measured by ELISA. The levels of ROS (D, *n* = 5) were assessed using the DCFDA assay. Data are given as scatter dot plots with the mean + SEM, Kruskal–Wallis (Benjamini–Hochberg FDR; A–C) or one-way ANOVA (Holm–Sidak post hoc; D), *⁣*^*∗*^*p* < 0.05, *⁣*^*∗*^ indicating statistical significance compared to LPS treatment.

The recruitment of neutrophils is orchestrated by a network of inflammatory signals, including TNF-α, IL-6, IL-10, and ROS, which collectively drive the inflammatory cascade during different pathological events. Our findings demonstrate that pretreatment of murine PMNs with 4-PBA prior to LPS stimulation leads to a significant reduction in both pro- (e.g., TNF-α, IL-6, ROS) and anti-inflammatory (e.g., IL-10) mediators, thereby promoting an overall anti-inflammatory phenotype (Figure 2).

### 4.3. 4-PBA Triggers Altered Inflammatory Responses in mPLMECs

**Figure 3:**
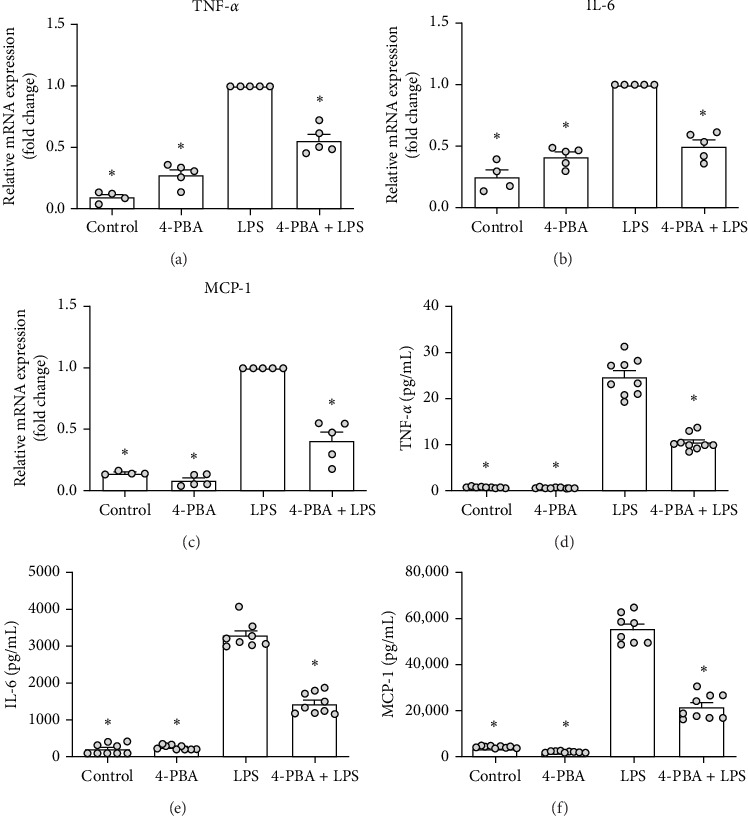
Effects of 4-PBA on the inflammatory cascade in mPLMECs in vitro. Mouse PLMECs were stimulated with 4-PBA, LPS, or a combination of 4-PBA and LPS as described. RNAs were collected 4 h posttreatment, and cDNA synthesis was performed for subsequent real-time PCR analysis of gene expression levels of TNF-α (A), IL-6 (B), and MCP-1 (C) (*n* = 4–5; LPS-treated cells were set to 1.0). Supernatants were harvested 12 h after the treatment to measure cytokine levels of TNF-α (D, *n* = 9), IL-6 (E, *n* = 9), and MCP-1 (F, *n* = 8–9) using ELISA. Data are given as scatter dot plots with the mean + SEM, one-way ANOVA (Holm–Sidak post hoc; A–C) or Kruskal–Wallis (Benjamini–Hochberg FDR; D–F), *⁣*^*∗*^*p* < 0.05, *⁣*^*∗*^ indicating statistical significance compared to LPS treatment.

To investigate the impact of 4-PBA on the endothelial layer, we examined the expression of key inflammatory mediators, including TNF-α and IL-6, as prototypical pro-inflammatory cytokines, and MCP-1, a CC-chemokine family member whose release is supported by IL-6 and plays a central role in neutrophil recruitment. Using real-time polymerase chain reaction (RT-PCR), we found that 4-PBA treatment significantly reduced LPS-induced TNF-α, IL-6, and MCP-1 expression in murine pulmonary microvascular endothelial cells (Figure 3). In line with this, at the protein level we observed a similar trend, with decreased release of TNF-α, IL-6, and MCP-1, further supporting the anti-inflammatory effect of 4-PBA on endothelial cells (Figure 3D–F).

### 4.4. 4-PBA Alters the Recruitment Cascade of PMNs and mPLMECs in Vitro

Transmigration is a pivotal process in neutrophil recruitment to sites of inflammation. Building on previous findings, we sought to determine whether treatment with 4-PBA would reduce the transmigration of PMNs and influence the recruitment patterns of both PMNs and endothelial cells (mPLMECs).

**Figure 4:**
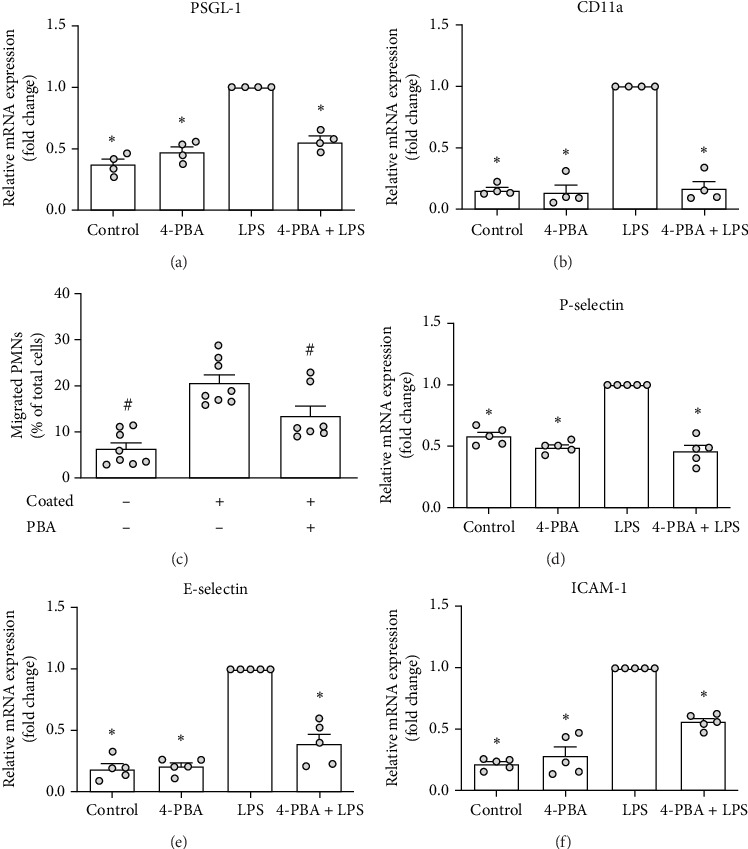
4-PBA modulates the migratory properties of bone marrow-derived PMNs and alters the expression of adhesion molecules in mPLMECs. Bone marrow-derived PMNs and mPLMECs were stimulated with 4-PBA, LPS, or a combination of 4-PBA and LPS as described. After 4 h, RNA was isolated, and gene expression of neutrophil surface molecules, including PSGL-1 (A, *n* = 4) and CD11a (B, *n* = 4), as well as endothelial adhesion molecules, such as P-selectin (D, *n* = 5), E-selectin (E, *n* = 5), and ICAM-1 (F, *n* = 5), were assessed by real-time PCR (LPS-treated cells were set to 1.0). The in vitro transmigration of PMNs (C, *n* = 7–8) in the presence or absence of a CXCL1/KC gradient (1 µg/mL), with or without 4-PBA incubation was evaluated. Data are given as scatter dot plots with the mean + SEM, analyzed by one-way ANOVA (Holm–Sidak post hoc; A, D–F) or Kruskal–Wallis (Benjamini–Hochberg FDR; B, C). Statistical significance is indicated by *p* < 0.05, with *⁣*^*∗*^ denoting significance compared to LPS treatment (A, B, D–F) and # denoting significance compared to CXCL1/KC-coated cells (C).

To evaluate this, we used RT-PCR to assess the expression of key molecules involved in neutrophil adhesion and transmigration. Specifically, we elaborated the expression of PSGL−1 and CD11a integrin on PMNs, as essential molecules contributing on initial tethering, rolling, firm adhesion and migration of neutrophils along the endothelial surface. The results demonstrated that 4-PBA treatment significantly reduced LPS-induced expression of PSGL-1 and CD11a on neutrophils in vitro (Figure 4A, B).

To further explore the functional implications of these findings, we conducted an in vitro transmigration assay. Consistent with the observed reduction in the expression of adhesion molecules, 4-PBA treatment markedly inhibited the transmigration of murine neutrophils in vitro (Figure 4C).

Additionally, we assessed the expression of P-selectin, E-selectin, and intercellular adhesion molecule (ICAM)−1 on endothelial cells, which are essential for facilitating neutrophil transmigration during inflammation. Our analysis revealed that 4-PBA treatment significantly reduced LPS-induced expression of these molecules in mPLMECs (Figure 4).

### 4.5. 4-PBA Regulates Neutrophil Activation via NF-κB Signaling Through the ERK1/2- IRE-1α Pathway

**Figure 5:**
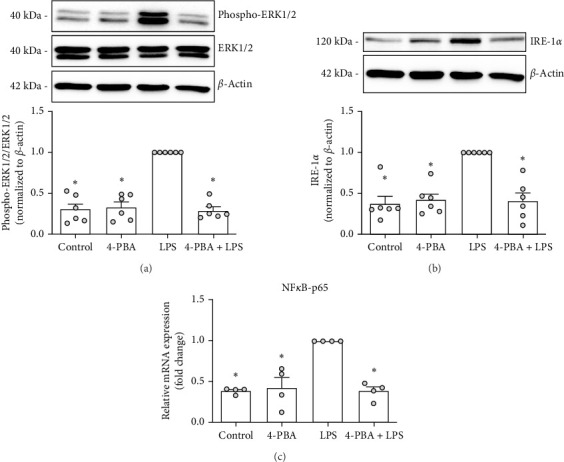
Molecular mechanism of 4-PBA treatment in bone marrow-derived PMNs. Bone marrow-derived PMNs were stimulated with 4-PBA, LPS, or a combination of 4-PBA and LPS as described. Lysates collected 12 h posttreatment were analyzed for protein expression of phospho-Erk1/2 (A, *n* = 6) and IRE-1α (B, *n* = 6) by western blotting and quantified (LPS-treated cells were set to 1.0). RNAs were collected 4 h posttreatment and analyzed for the gene expression of NF-κB-p65 (C, *n* = 4; LPS-treated cells were set to 1.0) using real-time PCR. Data are given as scatter dot plots with the mean + SEM, one-way ANOVA (Holm–Sidak post hoc; A, C) or Kruskal–Wallis (Benjamini–Hochberg FDR; B), *⁣*^*∗*^*p* < 0.05, *⁣*^*∗*^ indicating statistical significance compared to LPS treatment.

We next sought to identify the signaling molecules responsible for the reduced neutrophil recruitment and inflammation observed following 4-PBA treatment. Protein expression analysis of ERK 1/2, a key component of the mitogen-activated protein kinase (MAPK) pro-inflammatory pathway, as well as IRE-1α, a critical regulator of ER stress, revealed a significant reduction in their expression following 4-PBA treatment compared to the LPS-treated group (Figure 5A, B). It is well-known that IRE-1α activation can induce ERK 1/2 phosphorylation of I*κ*B, which leads to the nuclear translocation of nuclear factor kappa B (NF-κB). Consistent with this, our data demonstrate that 4-PBA treatment results in declined activation of NF-κB-p65 in PMNs in vitro (Figure 5C).

## 5. Discussion

Neutrophils as effector innate immune cells are critical players that are recruited to the inflamed tissue, where they either stimulate immune-protective and repair mechanisms or cause major tissue damage [41–43]. An emerging number of studies have reported that subclinical endotoxemia may trigger an elevated inflammatory state in innate immune cells, especially monocytes and neutrophils, involved in tissue infiltration during chronic inflammatory diseases [44–46]. If unchecked, PAMPs or other stressors arising from the tissues damage (damage-associated molecular patterns [DAMPs]) may trigger exacerbated activation of neutrophils, resulting in tissue injury [43, 47, 48]. Recent studies also demonstrate adaptive properties of polymorphonuclear cells, driving both antitumoral and antimicrobial actions [33–35, 49, 50]. On the functional level, neutrophil recruitment serves as a critical multistep cascade to regulate the inflammatory response and may drive the pathogenesis of vascular inflammation [51, 52].

Being able to modulate the balance between pro- and anti-inflammatory properties of neutrophils, as well as counteract hyperinflammation by dampening the extravasation of neutrophils, would represent a promising approach to tackle acute and chronic inflammatory disease. 4-PBA is a well-known chemical chaperone approved for clinical use in urea cycle disorders, a potent inhibitor of HDAC and ER stress that exhibits immune-suppressive effects [19–22, 53]. A recent study demonstrated that 4-PBA can effectively reprogram monocytes into an immune-resolving state, characterized by reduced adhesion and diminished inflammation, ultimately contributing to a decrease in atherosclerosis [54]. In parallel, Lin et al. [55] reported that 4-PBA induces a similar reprograming in neutrophils, promoting an anti-inflammatory phenotype marked by elevated expression of CD200R, an inhibitory receptor known to limit myeloid cell activation and support the resolution of inflammation. Similarly, our findings revealed that 4-PBA pretreatment significantly dampened neutrophil adherence, and interestingly, this effect was regulated in a developmental stage-dependent manner. Previous data from our group have shown that leukocyte recruitment, particularly the adhesive features of fetal leukocytes, increases with GA [56].

It is well-known that neutrophil recruitment cascade and inflammation are closely linked processes that play a pivotal role to support a proper immune response to infection or tissue injury, as well as in the resolution of inflammation. In addition, several studies have indicated that neutrophils promote their own recruitment by releasing a variety of inflammatory mediators, such as pro-inflammatory cytokines (e.g., TNF-α, IL-6) and ROS, which support neutrophil recruitment by inducing the production of chemokines (e.g., MCP-1) and modulating endothelial activation [5, 57, 58]. Investigation of the impact of 4-PBA on the inflammatory profile of neutrophils in vitro revealed significantly reduced levels of pro- and anti-inflammatory cytokines, such as TNF-α, IL-6 and IL-10. These data are in line with previous studies that have highlighted the pivotal role of these cytokines in promoting neutrophil and monocyte cell recruitment [59, 60]. While an earlier study by Sun et al. suggested that IL-10 may suppress the recruitment cascade of neutrophils, a growing body of evidence indicates that IL-10 promotes recruitment activities of neutrophils in a STAT3-dependent manner [61–64]. Furthermore, our data showed that 4-PBA supports a marked decrease of the ROS production in neutrophils in vitro. Likewise, several studies highlighted the pivotal role of ROS not exclusively limited to oxidative stress responses, but contributing also to neutrophil chemotaxis by modulating critical signaling inflammatory pathways like MAPKs affecting further the expression of adhesion molecules (e.g., P-selectin) in the endothelial compartment [65–67]. These results align with findings in mononuclear cells, such as monocytes, where reduced recruitment and inflammatory responses were also observed following 4-PBA treatment [25–27]. Furthermore, in a study by Zeng et al. [19] it was shown that 4-PBA dampens LPS-induced inflammation during acute lung injury (ALI) mainly through the inhibition of ER stress. Interestingly, the effect of 4-PBA treatment in LPS-injected mice triggered a distinct cytokine profile, with decreased levels of TNF-α consistent with its anti-inflammatory effects, however, it promoted a selective response with increased IL-6 and MCP-1 in the circulation. These contradictory findings might be explained by various cells present in the blood plasma that may exert different inflammatory reactions in response to 4-PBA. For instance, Zeng et al. [19] performed the study in alveolar epithelial cells. Moreover, in a study by Wang et al. [68] no impact of 4-PBA on the pro-inflammatory status of murine serum in vivo was found, but the authors interestingly reported an increased production of IL-35. Additionally, IL-35 is well-known to support the releases of both anti- (such as TGF-β) as well as pro-inflammatory mediators like IL-6 [69, 70].

To further understand the impact of 4-PBA on the neutrophil recruitment cascade, we performed comparable studies in the endothelial compartment. The role of endothelial cells in homing neutrophils is well described and attributed especially to the release of TNF-α, IL-6 and MCP-1 [71, 72]. As expected, mPLMECs pretreated with 4-PBA expressed diminished production levels of these inflammatory mediators. Prototypical pro-inflammatory cytokines, such as TNF-α and IL-6, are vastly known to promote the expression of several adhesion molecules (e.g., ICAM-1, VCAM-1) by endothelial cells [73, 74]. This, in turn, activates a self-amplifying feedback loop that encourages neutrophil binding, thereby promoting inflammatory events. In addition, IL-6 participates in neutrophil migration by inducing the production of chemokines, such as IL-8 and MCP-1 [75, 76]. MCP-1, released by endothelial cells, is known to orchestrate neutrophil migration under physiological and pathological circumstances [10, 11, 77]. As a result, the suppression of these inflammatory mediators by 4-PBA might explain impaired neutrophil recruitment observed in vivo.

A critical yet unresolved aspect of understanding the impact of 4-PBA on neutrophil recruitment is its role in the terminal step of extravasation – transmigration, and its implications on different adhesion molecules in neutrophils and endothelial cells. In response to inflammatory signals, endothelial cells become activated and upregulate the expression of various adhesion molecules, such as P-selectin, E-selectin, and ICAM-1, which facilitate the interaction between neutrophils and the endothelium, contributing to firm adhesion and thus enabling subsequent transmigration of neutrophils into inflamed tissues [7, 78]. Our data demonstrate that beside attenuated neutrophil migratory capacity in vitro, 4-PBA reduced the expression of essential adhesion molecules, particularly PSGL-1 and integrin αL (CD11a) on neutrophils, as well as their corresponding ligands, such as P-selectin, E-selectin and ICAM-1, on endothelial cells. Correspondingly, several reports have demonstrated that 4-PBA effectively attenuates monocyte recruitment byreducing ICAM-1 expression through the mechanistic target of rapamycin (mTOR) signaling pathway, ultimately contributing to a reduction in the pathogenesis of atherosclerosis [25, 54]. These findings suggest that 4-PBA interferes with both the neutrophil and endothelial compartments, likely through a concerted regulation of the recruitment cascade, thereby controlling excessive inflammation.

As a chemical chaperone and a potent inhibitor of histone deacetylase activities (HDACs), 4-PBA, rescues cells from ER stress by preventing the aggregation of misfolded proteins [79, 80]. During cellular stress or injury, ER stress responses are activated influencing a variety of signaling cascades, involving particularly MAPKs like ERK1/2 [81, 82]. Furthermore, HDACs are well-known enzymes that regulate gene expression and may be directly involved in alterations within the ERK1/2 signaling cascade, especially HDAC3, which is required for the activation of phosphatidylinositol 3-kinases (PI3Ks) and ERK1/2 [83]. Our data showed that 4-PBA treatment triggering diminished recruitment activity with declined inflammatory state is particularly regulated by ERK1/2 pathway. A close interplay between ERK1/2 activation and UPR by ER stress is evident [81]. IRE-1α, as the most prominent transmembrane protein of ER, serves as critical sensor mediating signal transmission during UPR activation [84]. An increasing number of studies have shown that IRE-1α can also activate various MAPKs, leading to the subsequent activation of NF-κB, particularly NF-κB-p65, which drives the regulation of different inflammatory responses [85, 86]. Similarly, our data show that reduced inflammation and impaired neutrophil recruitment induced by 4-PBA are primarily regulated by the reduced activation of NF-κB-p65, mediated by IRE-1α/ERK1/2 pathway.

Overall, we were able to elucidate the role of 4-PBA in regulating the neutrophil recruitment cascade supported by distinct release of several inflammatory mediators by neutrophils and endothelial cells. These data suggest that 4-PBA may exert anti-inflammatory effects by limiting excessive neutrophil infiltration into inflamed tissues via a downregulation of IRE-1α/ERK1/2/NF-κB-p65 signaling pathway. It is important to emphasize that by impairing the migratory abilities of neutrophils and other immune cells, 4-PBA as an ER stress inhibitor and ammonia scavenger might hold significant potential in moderating prolonged inflammatory responses, with implications for diseases characterized by aberrant neutrophil infiltration and chronic inflammation [41, 87–90]. Further studies are warranted to explore the therapeutic potential of 4-PBA in inflammatory disorders and its impact on other immune cells, potentially paving the way for novel ER stress-targeted anti-inflammatory strategies.

## Data Availability

The datasets generated and/or analyzed during the current study are available from the corresponding author upon reasonable request.

## Supporting Information

Supporting Information Figure S1: Purity analysis and cytotoxicity of PMNs following 4-PBA treatment. Representative flow cytometry plots illustrating the purity of bone marrow-derived PMNs isolated via density gradient centrifugation. Live cells (PI-negative; BD Pharmingen Propidium Iodide Staining Solution, #556463, BD Biosciences, Heidelberg, Germany) were then gated, and neutrophil purity (A) was confirmed by the expression of Ly-6G (BD Pharmingen FITC Rat anti-Mouse Ly-6 G, #561105, BD Biosciences, Heidelberg, Germany) and CD11b (APC anti-mouse/human CD11b Antibody, #101211, BioLegend, San Diego, USA), with neutrophils gated as Ly6G+CD11b+ cells. After isolation, PMNs were treated with 4-PBA, LPS, or a combination of both, as described. The cytotoxicity (B, *n* = 4) of 4-PBA was evaluated using commercial cytotoxicity kits, whereas cell viability (C, *n* = 3) was assessed using the MTT assay (control groups assigned as 100%). Data are given as scatter dot plots with the mean + SEM. Figure S2: 4-PBA treatment supports a distinct inflammatory profile in murine blood plasma. C57BL/6 mice were treated as described above with either 0.9% NaCl (control), 4-PBA (25 mg/kg), LPS (0.25 mg/kg) or a combination of 4-PBA and LPS (25 mg/kg 4-PBA pretreatment, 0.25 mg/kg LPS) for 24 h. Blood plasma was collected and assayed for the cytokine levels of TNF-*α* (A, *n* = 4–7), IL-6 (A, *n* = 5–9), MCP-1 (B, *n* = 6–9) and IL-10 (C, *n* = 6–9) using ELISA, and the data were normalized to the corresponding total protein concentrations. Data are given as scatter dot plots with the mean + SEM, Kruskal–Wallis (Benjamini–Hochberg FDR), *⁣*^*∗*^*p* < 0.05, *⁣*^*∗*^ indicating statistical significance compared to the LPS-treated group.
