# Supplementary figures and images for: 4-Phenyl Butyric Acid (4-PBA) Suppresses Neutrophil Recruitment in a Murine Model of Acute Perinatal Inflammation
Source: J Immunol Res. 2025 Jul 7;2025:2438058. doi: 10.1155/jimr/2438058 (PMC12259320; doi:10.1155/jimr/2438058)

**Supplementary Figures**

**Suppl. Figure 1**


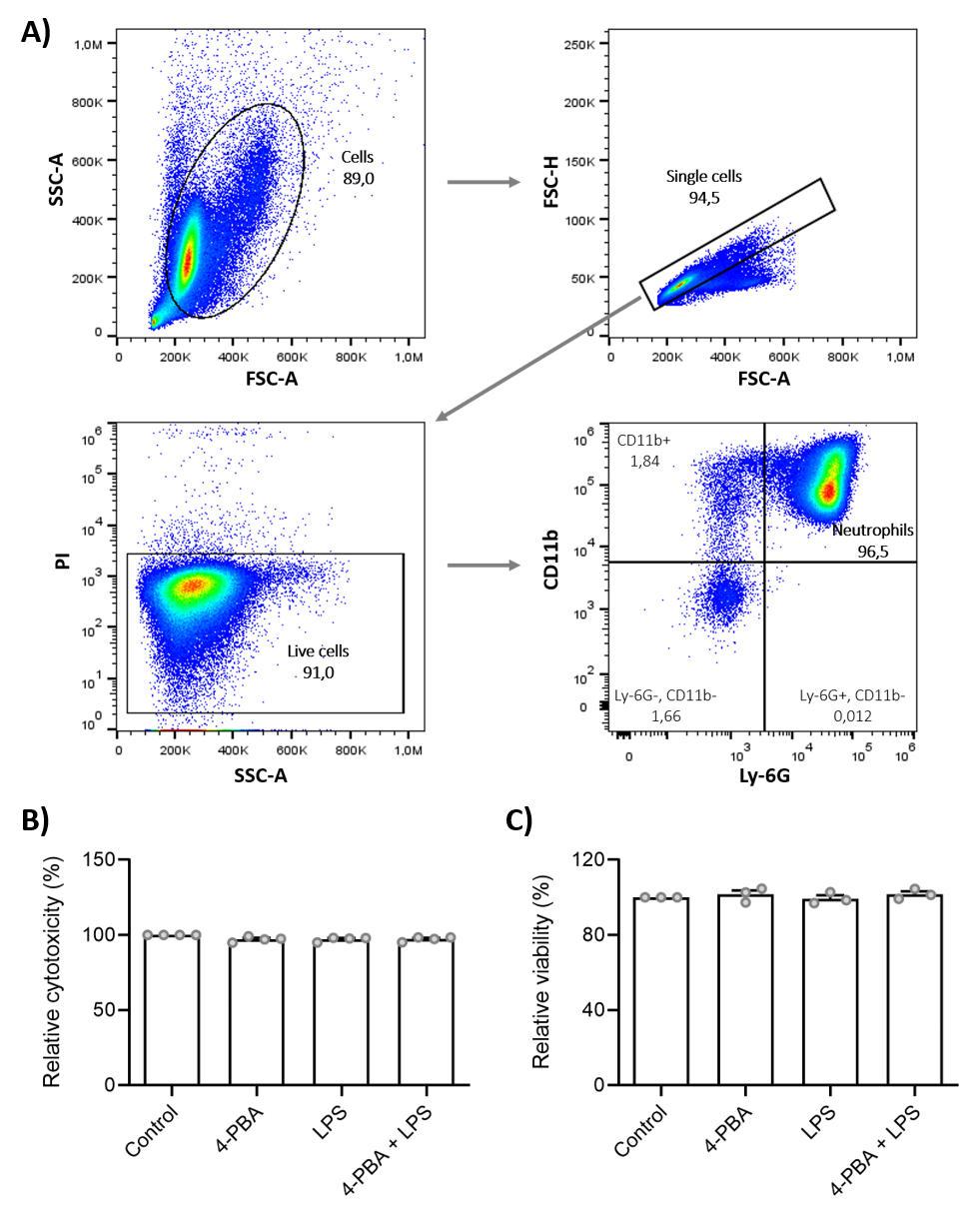


**Suppl. Figure 2**


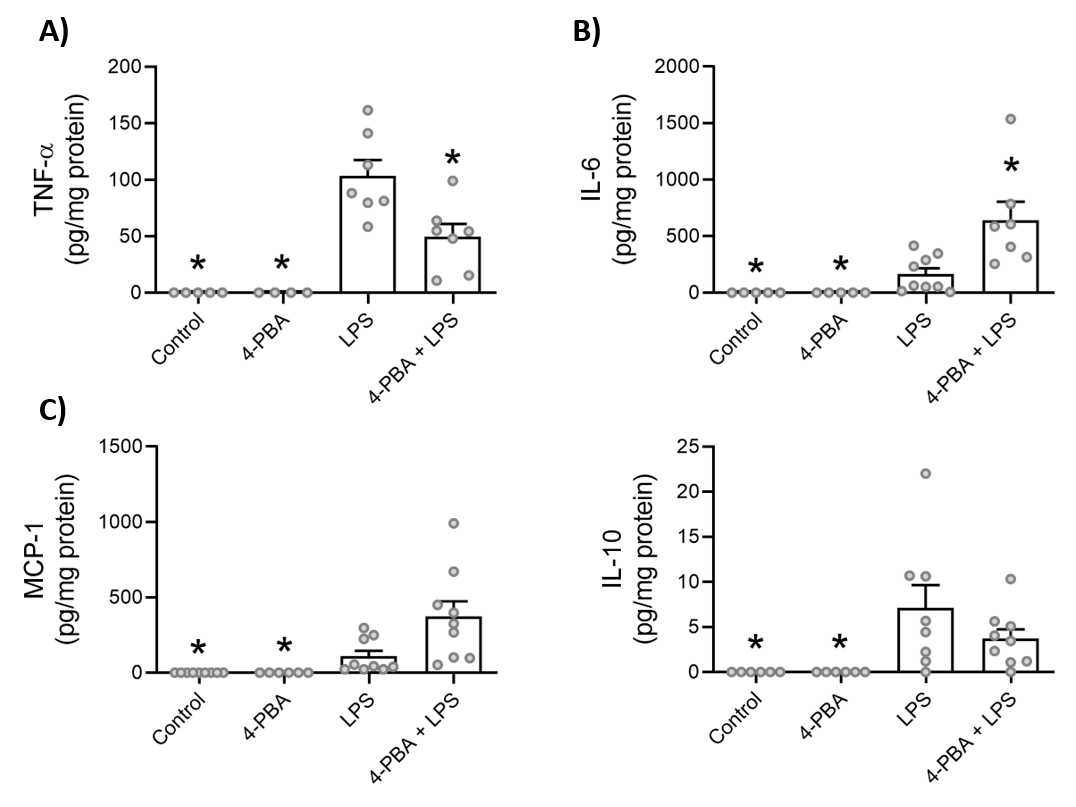

Supplement: Supporting Information — Figure S1: Purity analysis and cytotoxicity of PMNs following 4-PBA treatment. Representative flow cytometry plots illustrating the purity of bone marrow-derived PMNs isolated via density gradient centrifugation. Live cells (PI-negative; BD Pharmingen Propidium Iodide Staining Solution, #556463, BD Biosciences, Heidelberg, Germany) were then gated, and neutrophil purity (A) was confirmed by the expression of Ly-6G (BD Pharmingen FITC Rat anti-Mouse Ly-6 G, #561105, BD Biosciences, Heidelberg, Germany) and CD11b (APC anti-mouse/human CD11b Antibody, #101211, BioLegend, San Diego, USA), with neutrophils gated as Ly6G+CD11b+ cells. After isolation, PMNs were treated with 4-PBA, LPS, or a combination of both, as described. The cytotoxicity (B, n = 4) of 4-PBA was evaluated using commercial cytotoxicity kits, whereas cell viability (C, n = 3) was assessed using the MTT assay (control groups assigned as 100%). Data are given as scatter dot plots with the mean + SEM. Figure S2: 4-PBA treatment supports a distinct inflammatory profile in murine blood plasma. C57BL/6 mice were treated as described above with either 0.9% NaCl (control), 4-PBA (25 mg/kg), LPS (0.25 mg/kg) or a combination of 4-PBA and LPS (25 mg/kg 4-PBA pretreatment, 0.25 mg/kg LPS) for 24 h. Blood plasma was collected and assayed for the cytokine levels of TNF-α (A, n = 4–7), IL-6 (A, n = 5–9), MCP-1 (B, n = 6–9) and IL-10 (C, n = 6–9) using ELISA, and the data were normalized to the corresponding total protein concentrations. Data are given as scatter dot plots with the mean + SEM, Kruskal–Wallis (Benjamini–Hochberg FDR), ⁣∗p < 0.05, ⁣∗ indicating statistical significance compared to the LPS-treated group. [file 2438058.f1.docx]
